# Draft Genome Sequence of the *Aeromonas diversa* Type Strain

**DOI:** 10.1128/genomeA.00330-13

**Published:** 2013-06-06

**Authors:** Maribel Farfán, Nino Spataro, Ariadna Sanglas, Vicenta Albarral, J. Gaspar Lorén, Elena Bosch, M. Carmen Fusté

**Affiliations:** Departament de Microbiologia i Parasitologia Sanitàries, Facultat de Farmàcia, Universitat de Barcelona, Barcelona, Spaina; Institute of Evolutionary Biology (CSIC-UPF), Department of Experimental and Health Sciences, Universitat Pompeu Fabra, Barcelona, Spainc; Institut de Recerca de la Biodiversitat (IRBio), Universitat de Barcelona, Barcelona, Spainb

## Abstract

We present here the first genome sequence of the *Aeromonas diversa* type strain (CECT 4254^T^). This strain was isolated from the leg wound of a patient in New Orleans (Louisiana) and was originally described as enteric group 501 and distinguished from *A. schubertii* by DNA-DNA hybridization and phenotypical characterization.

## GENOME ANNOUNCEMENT

The genus *Aeromonas* Stanier 1943, 213^AL^, belongs to the family *Aeromonadaceae* within the class *Gammaproteobacteria* (1). Aeromonads are autochthonous inhabitants of aquatic environments, including chlorinated and polluted waters, although they can also be isolated from a wide variety of environmental and clinical sources. They cause infections in vertebrates and invertebrates, such as frogs, birds, various fish species, and domestic animals. In recent years, some authors have considered *Aeromonas* as an emergent pathogen in humans, producing intestinal and extra-intestinal diseases. Aeromonads are facultative anaerobic chemo-organotrophs capable of anaerobic nitrate respiration and dissimilatory metal reduction (1). *Aeromonas* sp. 2478-85 Hickman-Brenner et al. 1988 (CDC 2478-85, ATCC 43946, CECT 4254) is the type strain of *Aeromonas diversa* (2). This strain was isolated from the leg wound of a patient of the Charity Hospital in New Orleans (Louisiana), initially designated enteric group 501 (3) and then in 1991 proposed to be DNA hybridization group 13 (HG13) (4).

The draft genome sequence of the *A. diversa* type strain was obtained with a shotgun strategy using Roche 454 sequencing technology. A total of 158,564 reads with an average length of 442 nucleotides (15× coverage) were *de novo* assembled by a combined strategy (Newbler *de novo* and Velvet *de novo*) using the AMOS package 3.1.0. A total of 104 contigs, 101 of >1 kb in length, were constructed, with an N_50_ of 64,780 bp; the largest contig assembled measured 227,398 bp and the calculated draft genome size was 4.02 Mb, which is slightly smaller than the other *Aeromonas* genomes reported to date (ranging from 4.3 to 4.97 Mb) (5–13). The G+C mole percentage was 61.7.

The gene prediction and protein annotation were performed by applying the NCBI Prokaryotic Genomes Automatic Annotation Pipeline (http://www.ncbi.nlm.nih.gov/genomes/static/Pipeline.html) on the assembled contigs. A total of 3,709 protein-coding sequences were identified, together with 68 tRNA genes and 6 rRNA genes. Protein annotation using the VFDB database (http://www.mgc.ac.cn/VFs/) of virulent factors for bacterial pathogens detected seven putative virulence factors, including a gene involved in ferric uptake (*hemE*), a fosfoheptose isomerase gene related to lipopolysaccharide (LPS) biosynthesis (*gmhA*), three genes (*cheW, fliN* and *cheY*) involved in flagellar motor switch component signal transmission, and two type III secretion system genes (transport H^+^ ATPase gene, *yscR*). Further studies on these virulence-associated proteins will enhance our understanding of *Aeromonas* infections in humans.

### Nucleotide sequence accession numbers.

This whole-genome shotgun project has been deposited at DDBJ/EMBL/GenBank under the accession number APVG00000000. The version described in this paper is the first version, APVG01000000.
